# Effect of Supplementation with Trimethylglycine (Betaine) and/or Vitamins on Semen Quality, Fertility, Antioxidant Status, DNA Repair and Welfare of Roosters Exposed to Chronic Heat Stress

**DOI:** 10.3390/ani9080547

**Published:** 2019-08-12

**Authors:** Youssef A. Attia, Asmaa Sh. El-Naggar, Bahaa M. Abou-Shehema, Ahmed A. Abdella

**Affiliations:** 1 Arid Land Agriculture Department, Faculty of Meteorology, Environment and Arid Land Agriculture, King Abdulaziz University, Jeddah 21589, Saudi Arabia; 2 Animal and Poultry Production Department, Faculty of Agriculture, Damanhour University, Damanhour 22713, Egypt; 3 Department of Poultry Nutrition, Animal production Research Institute, Agriculture Research Center, Ministry of Agriculture and Land Reclamation, Alexandria 21917, Egypt

**Keywords:** betaine, vitamin C, vitamin E, heat stress, roosters, semen quality, blood constituents

## Abstract

**Simple Summary:**

Semen, reproductive traits, and the welfare of males are negatively affected by environmental stressors. Stress-alleviating agents, such as vitamins and osmoregulators, may improve semen quality, seminal and blood plasma constituents, antioxidants’ status, and the welfare of roosters exposed to chronic heat stress (CHS). It has been shown that betaine (Bet) may be a useful tool for improving the reproductive traits of roosters exposed to CHS, and may have comparable effects to vitamin C and/or E, thus improving the breeding strategy.

**Abstract:**

In this study, we investigated the influence of betaine (Bet, 1000 mg/kg), with or without vitamin C (VC, 200 mg/kg ascorbic acid) and/or vitamin E (VE, 150 mg/kg α-tocopherol acetate) on semen quality, seminal and blood plasma constituents, antioxidants’ status, DNA repair, and the welfare of chronic heat stress (CHS)-exposed roosters. A total of 54 roosters were divided into six groups of nine replicates. One group was kept under thermoneutral conditions, whereas the other five were kept under CHS. One of the five groups served as an unsupplemented CHS group, and was fed with a basal diet. The other four CHS groups were supplemented with Bet, Bet + VC, Bet + VE, and Bet + VC + VE, respectively. Our data indicate that supplementation with Bet, Bet + VC, Bet + VE, and Bet + VC + VE, resulted in complete recovery of the CHS effect on sperm concentration and livability, semen pH, and fertility compared to the thermoneutral group. Seminal plasma total antioxidant capacity (TAC) was significantly (*p* < 0.05) increased with Bet, with or without vitamins, compared to the thermoneutral and CHS groups. Urea and blood plasma malondialdehyde (MDA) were totally recovered with Bet, with or without vitamin treatments. Both the jejunum and ileum DNA were partially recovered following Bet, with or without vitamin supplementation. In conclusion, Bet, at 1000 mg/kg feed, may be a useful agent for increasing semen quality, fertility, welfare, and to improve the breeding strategy of breeder males in hot climates.

## 1. Introduction

Oxidative stress plays a key role in sperm function and motility, cell quality, and fertility, since lipid peroxidation increases under chronic heat stress (CHS), particularly when the temperature exceeds 27 °C [1]. Heat stress negatively influences testis functions [2,3], causes DNA damage [2,4], lowers the quality of semen [5], and reduces animal wellbeing [2]. In addition, HS causes abnormal mitotic division during spermatogenesis and produces abnormal spermatozoa that may not complete the fertilization process [6].

Avian spermatozoa are rich in polyunsaturated fatty acids (PUFA) and are, therefore, subjective to reactive oxygen species (ROS), causing male infertility [7]. Therefore, antioxidants are essential for avoiding male infertility by limiting free radical production [1,8]. Betaine (Bet) is a trimethylglycine produced by choline oxidation [9], and it is implicated in methionine and choline sparing [10,11], in fat distribution, and immune responses [12]. Bet has also been found to improve tolerance to stress [9,13], but studies on the reproduction of roosters remain limited. However, a study conducted in boars is available [14], which indicates that dietary 0.63 and 1.26% Bet elevated the amount of sperm compared to the control animals. Adding 200 mg/kg of Bet to the diet decreased the mortality of heat-stressed chickens; enhanced their immunity and health status [15]; and increased their antioxidant status, such as glutathione peroxidase (GPx) and superoxide dismutase (SOD), while decreasing malondialdhyde (MDA) in blood serum [16] and in breast muscle tissue [17].

Vitamin C (VC, ascorbic acid) has antioxidant activity that protects animals under stress conditions [18], and animals can synthesize VC under normal conditions. However, adding VC improves performance, immunological status, and welfare of the animals exposed to HS [8,18]. Chickens require VC to metabolize minerals and amino acids, as well as for the activity of leukocytes and 1,25-dihydroxy vitamin D, collagen, the regulation of body temperature, corticosterone secretion, adrenaline biosynthesis [1], and testosterone synthesis [19]. Heat stress impairs ascorbic acid absorption and increases the need for VC [20]. Moreover, the impact of VC on semen quality is quite inconsistent [6] and dose-specific [1]. This may be due to the heat destruction of VC [21].

Alpha-tocopherol (vitamin E, VE) is a natural antioxidant and an excellent biological chain-breaking antioxidant that is involved in tissue protection from oxidative damage and ROS [22]. A VE diet that exceeds 15 mg/kg (100–150 mg/kg) [23] was found to boost the semen quality and fertilizing ability of roosters [24,25,26] and boars [27]. Furthermore, a 400 mg/day dose of VE was recommended for the treatment of male infertility in men [28]. Therefore, dietary supplementation with VE is essential for compacting the adverse effects of HS on the lipid peroxidation of spermatozoa cell membranes [22], and it enhances plasma cells, lymphocytes, and macrophages against oxidative damage [29]. The influence of HS and the combined effect of different antioxidant agents working by different modes of action to relieve the negative impact of CHS are scarce in the available literature and particularly in the nutrition of roosters. Currently, no studies address the effects of Bet alone or in combination with antioxidant vitamins on semen quality, reproductive and breeding strategy of males to elucidate their synergetic effect on modes of action. Hoehler and Marquardt [30] indicated that the in vivo antioxidant influence of VE might be greater than that of VC. Supplementation with Bet, VC, or a combination of both, resulted in significantly increased total protein and globulin of laying hens exposed to HS, while serum triglyceride, total cholesterol, HDL-cholesterol, and glucose were significantly decreased in comparison to the control group [31]. In addition, Attia et al. [15] found that Bet and VC have similar effects on production traits, metabolism, blood constituents, and the wellbeing of laying hens exposed to chronic heat stress (CHS).

Therefore, the aim of this study was to test the influence of the HS and Bet (Bet + VC, Bet + VE, and Bet + VC + VE) on the quality of semen, seminal and blood plasma metabolites, reproductive performance, the wellbeing, and the DNA of intestinal segments of roosters.

## 2. Materials and Methods

### 2.1. Animals and Treatments

The protocol for this experiment was approved by the scientific committee of the Animal Production Research Institute under the registration code no: 9-2-4-3-10-1. The committee recommended that care and handling of the animals maintains their rights, welfare, with minimal stress, according to International Guidelines for research involving animals (Directive2010/63/EU).

A total of 54, 32-week-old male Mandarah (a dual-purpose breed) chickens, with a similar initial body weight were distributed randomly among six treatment groups of nine males during weeks 32–52 of age. Each male served as a replicate, and was individually housed in galvanized wire cages in batteries with standard dimensions (30 × 50 × 60 cm) in an environmentally controlled lightproof house (close system; controlled for temperature, humidity, and light). Each cage was provided with a manual feeder and nipple waters. Chickens were offered free access to mash diets and water throughout the experimental period. The experimental diets’ chemical composition was done according to [32].

The health care, housing condition, heat stress protocol, indoor and outdoor temperature, relative humidity (RH), and light schedule were similar to those reported in [15]. The roosters were reared (indoor) either at an optimum temperature of 22–24 °C, with RH of 45–55%, serving as the thermoneutral group (positive control). They were fed with a basal diet (Table 1) or under CHS (38 ± 1 °C; 55–65% RH) for three successive days a week, from 11.00 a.m. to 15.00 p.m., and returned to the thermoneutral condition thereafter. Roosters under CHS were divided into four groups: roosters kept under CHS and fed with a basal diet without additional Bet, Bet + VC, Bet + VE, and Bet + VC + VE serving as the CHS negative control. The Bet group, roosters kept under CHS and fed with a basal diet supplemented with 1000 mg/kg of Bet (natural Betafin^®^ S4 contain 93% dry Bet, Danisco Animal Nutrition, Marlborough, UK). The Bet +VC group, roosters kept under CHS and fed a basal diet supplemented with 1000 mg/kg betaine + 200 mg/kg ascorbic acid (l-ascorbic acid; a heat stabilized product, Hoffmann-La Roche, Basel, Switzerland). The Bet+VE group, roosters kept under CHS and fed a basal diet supplemented with 1000 mg/kg betaine + 150 mg/kg α-tocopherol acetate (VE; α-tocopherol acetate, Hoffmann-La Roche, Switzerland). The Bet+VC+VE group, roosters kept under CHS and fed with a basal diet supplemented with 1000 mg/kg betaine + with 200 mg/kg ascorbic acid + 150 mg/kg α-tocopherol acetate (VC + VE).

### 2.2. Data Collection

Roosters were individually weighted at 32 and 52 weeks of age, body weight gain was calculated from the difference between the initial and final body weight. Daily feed intake (g/bird) and mortality were recorded for each replicate.

Semen was collected weekly from all roosters after 10 weeks of treatments at week 42 of age, and maintained for another 10 weeks to determine the semen’s quality as outlined by Attia et al. [33]. Semen collection was performed under the procedure of abdominal massage. The internal temperature of the semen at the time of collection was kept in the range of 41–44 °C using a water bath (at 37 °C). Semen samples were then transferred immediately after collection to the laboratory to determine spermatozoa quality. Moreover, special attention was given to protect semen from cold shocks and direct light. Throughout the course of semen collection, time, place of collection, and collector were kept constant.

At weeks 44, 48, and 52 of age, the roosters’ fertility was evaluated. Semen samples were artificially collected by abdominal massage and used for the artificial insemination of hens. Semen was used after 1:1 dilution using a 0.9% saline solution as a diluent [34]. The semen of each male was used to inseminate 10 hens. Each hen was inseminated with 0.5 mL of semen over two successive days. After two days of insemination, the eggs were collected for ten days, and stored at room temperature (22–24 °C with 45–55% RH), incubated (37.6°C, 55% RH), and hatched (36.8°C, 65% RH) in an automatic incubator. Fertility was calculated by dividing the number of fertile eggs by the total eggs set.

At 52 weeks of age, blood samples (5 mL) were withdrawn from the wing vein after each treatment. Blood samples (*n* = 5) after each treatment were collected heparin (an anticoagulant agent) tubes in the morning from the overnight-fasted roosters. Blood plasma and seminal plasma were obtained by blood and semen centrifugation at 1500× g for 20 min, and kept at −20 °C until the analysis was performed [33].

Plasma and seminal plasma metabolites, seminal and blood plasma total antioxidant capacity (TAC), and malondialdhyde (MDA) were determined using a diagnostic kit and following the manufacture’s recommendation (Diamond diagnostics, 23 EL-Montazah St. Heliopolis, Cairo, Egypt, http://www.diamonddiagnostics.com). Blood plasma creatinine was measured using special kits delivered from N.S. BIOTEC (http://www.nsbiotec.com). The aspartate aminotransferase (AST) and alanine aminotransferase (ALT) as (U/L) in the blood and seminal plasma were determined using commercial kits produced by the Pasteur Lab (http://www.pasteurvetlab.com). Blood plasma alkaline phosphatase was measured according to the method by Yan et al. [35]. Seminal plasma α-, β-, and γ-globulin were determined using commercial ELISA according to the method by Bianchi et al. [36].

Blood hematological parameters, such as hemoglobin (Hgb; %), were determined according to the method by Tietz [37]. Red blood cells (RBCs) were counted on a bright line hemocytometer using a light microscope at 400× magnification according to the methods by Helper [38], and Hawkeye and Dennett [39]. Packed cell volume (PCV; %) was measured according to the method by Wintrobe [40]. The mean cell volume (MCV), the mean cell hemoglobin (MCH), and the mean cell hemoglobin concentration (MCHC), were estimated as absolute values as reported by Attia et al. [34]. The phagocytic activity and index were determined according to the method by Kawahara [41]. White blood cells (WBCs) were assessed according to the methods by Helper [38], and Dennett [39], using a light microscope at 100× magnification. The blood film was prepared according to the method by Lucky [42] to determine different leucocytes.

High molecular weight DNA was extracted from intestinal parts (jejunum and ileum) according to the method by Sambrook et al. [43], with some modification according to Abdel-Fattah [44]. The DNA concentration was estimated from the optical density (O.D.) reading of a UV spectrophotometer at a 260 nm wave-length (1.0 O.D. = 50 µg DNA/mL of solution), according to the method by Charles [45].

### 2.3. Statistical Analysis

Data were tested using the GLM procedure published by SAS^®^ (SAS Institute, Cary, NC, USA) [46], using one-way ANOVA according to the following model: yij = µ + τj + εij, where µ = the general mean, τj = the effect of treatment, and εij = the experimental error. A *p* ≤ 0.05 value of significance for the student Newman Klaus test was used for testing mean differences among the experimental groups. Prior to the analyses, all the percentages were subjected to logarithmic transformation to normalize data distribution.

## 3. Results

Overall, neither diseases symptoms nor mortality occurred throughout the experimental period.

The initial roosters’ body weights (BW) were not significantly (*p* > 0.001) different, while their BW changes and feed intake significantly decreased with the increase in the level of CHS, as indicated in Table 2. Body weight changes were completely resorted with high supplementations, except for Bet + VC, which caused partial recovery. All Bet-supplemented groups produced similar partial recovery in feed intake.

Table 2 indicates that the group on CHS without antioxidant addition significantly decreased semen’s physical characteristics. The ejaculate volume, sperm concentrate, concentrate/ejaculate, sperm motility %, sperm livability %, total live sperm/ejaculate, semen quality factor, and fertility for CHS groups were significantly decreased (*p* > 0.001) by 23.2, 23.6, 40.8, 13.1, 9.3, 46.1, 46.0, and 17.9%, respectively, compared to the thermoneutral control group. However, sperm mortality (%) and pH significantly increased (*p* > 0.001) by 62.8 and 3.7%, respectively.

The antioxidants, either individually or combined, induced complete recovery in sperm concentration, livability, pH, and fertility. Moreover, supplementation of Bet + VC + VE restored ejaculation volume, concentration/ejaculate, sperm motility, total live sperm/ejaculate, and the semen’s quality factor to the thermoneutral group, but differences among these groups and Bet or Bet + VE groups were not significant.

Groups of CHS roosters without antioxidants had significantly impaired total protein, globulin, AST, ALT, and TAC in seminal plasma compared to the thermoneutral group (Table 2). Antioxidants significantly (*p* > 0.001) restored total protein, globulin, AST, ALT, TAC, and MDA, indicating no significant difference from the thermoneutral group. When Bet + VC + VE were added, this caused an increase in γ-globulin compared to the group on Bet alone. Antioxidant groups showed increased TAC in comparison to both the thermoneutral and CHS groups. The different treatments did not cause any significant effects on plasma albumin, albumin/globulin ratio α- and β-globulin, and AST/ALT ratio.

Results in Table 3 show that the CHS group without antioxidants had significantly impaired RBCs, Hgb, PCV %, pH, PA, and PI compared to the thermoneutral group, the values decreased by 17.8, 20.6, 19.0, 2.8,2 and 28.9%, respectively. When antioxidants were added, a complete recovery of all hematological traits occurred except for PI, which was recovered only when the three additives were combined. Furthermore, PA was partially recovered similarly due to the different antioxidant supplementations. There were no significant effects from the different treatments on MCV, MCH, and MCHC.

White blood cell counts (−14.5%), lymphocytes (−9.4%), heterophile (+15.4%), H/L ratio as the welfare index (+22.9%), were negatively affected under the CHS exposure without antioxidants condition compared to the thermoneutral group. The addition of Bet + VC, Bet + VE, and Bet + VC + VE completely recovered the negative effects of HS on the WBC parameters and index of wellbeing (H/L). Monocyte, basophil, and eosinophil percentages were insignificantly affected by the different treatments.

Results in Table 4 show that CHS caused a significant impairment in plasma glucose, protein profiles except for plasma globulin, lipid profiles, and the liver function index, renal function index except for plasma urea/creatinine ratio, TAC, and MDA.

The addition of Bet alone or in combination with vitamins caused similar complete recovery in plasma glucose, the A/G ratio, total lipids, cholesterol, AST, ALT, the AST/ALT ratio, urea, and TAC. Instead, Bet and Bet + VC + VE caused a complete recovery in plasma total protein, and the combination of Bet + VC, Bet + VE, and Bet + VC + VE groups resulted in a complete recovery in plasma creatinine. The combination of Bet + VE and Bet + VC + VE completely recovered TAC, and had stronger effect than Bet alone. Furthermore, the three agents had a stronger effect compared to Bet + VC. Bet treatments caused a complete recovery in MDA, and the combined agents resulted in a stronger effect compared to Bet alone.

Antioxidant supplementation showed a partial recovery in plasma albumin with Bet and Bet + VC + VE, and resulted in a stronger recovery compared to the Bet + VE group. In addition, plasma triglyceride was partially recovered similarly due to the different antioxidants. Plasma alkaline phosphatase was partially recovered by the antioxidants. However, Bet + VE and Bet + VC + VE produced stronger effects compared to Bet and Bet + VC. Furthermore, the combination of the three agents resulted in a stronger effect compared to Bet + VE. The combination of Bet + VC, Bet + VE, and Bet + VC + VE showed a synergetic effect on TAC and MDA, which surpassed the effect of Bet and the thermoneutral groups.

Jejunum and ileum DNA concentrations were significantly affected, negatively by HS and positively by antioxidants (Table 4). The CHS treatment without antioxidants significantly decreased jejunum and ileum DNA concentrations by 10.2% and 19.8%, respectively, compared to the thermoneutral group. Antioxidants significantly increased DNA concentrations in the jejunum and ileum compared to the CHS group. With Bet and Bet + VE surpassing the other antioxidant groups to result in complete recovery of ileum DNA only.

## 4. Discussion

Heat stress negatively affects both animal and human welfare. The use of Bet with or without vitamin fortifications to relief the adverse influence of HS is an essential tool in human and animal nutrition. In this study, we demonstrate that HS negatively affects fertility and semen quality, and that Bet supplementation alone showed promising relieving effects. The negative effects of CHS caused an increase in the H/L ratio in roosters exposed to HS, suggesting their low [1,6]. Similarly, H/L appears to be a more reliable indicator for determining stress and welfare in poultry [47], which has negative effects on immunity and disease resistance [18,48]. The reduction in TAC and elevation in MDA confirmed the adverse effects of CHS on antioxidant conditions [49,50]. In addition, blood biochemistry and immunity indices were negatively affected herein by HS. More specifically, we observed a decrease in seminal and blood plasma total protein, and blood plasma albumin (nonspecific immune protein), γ-globulin (innate immunity), PA and PI (nonspecific immunity), WBCs and lymphocyte (cell mediated immunity), and DNA of the intestinal segments. In addition, RBCs, Hgb concentration, and PCV were adversely affected due to CHS, showing low animal welfare and health. This could be attributed to the decline in RBCs, which reduces oxygen uptake, resulting in less metabolic heat loss. In accordance with the present results, studies described in References [51] and [52] show that HS decreases Hgb and PCV while increasing blood pH. This was in agreement with the low availability of essential nutrients for DNA synthesis, functions, and repairs [53,54] and increased water intake [48].

Our study shows a decrease in the semen quality and fertility of roosters exposed to HS, and this was associated with the reduction in this groups feed intake. A HS > 31 °C is known to depress rooster sperm motility, viability, and fertilization potential [6]. This could be attributed to the decrease in sperm motility and the number of spermatozoa stored in the sperm host gland of hens [1]. In addition, HS can have negative effects on testosterone, causing hypertrophy and weakening of Leydig cell function [55]. The sperm’s damaged DNA can determine abnormal spermatozoa, which could cause low male fertility and subsequently fewer surviving embryos [4,56,57]. Even if the sperm can fertilize eggs normally, embryos that have received an injured paternal genome could die, causing poor performance [58]. The biochemical and immunological change in blood and seminal plasma was reflected in lower semen quality and reproductive efficiency, showing low breeding strategy under CHS.

In the present study, supplementing Bet alone caused a complete recovery in BW, sperm concentration, motility, livability of sperm, semen pH, fertility, seminal plasma total protein, globulin, seminal plasma AST and ALT, RBCs, Hgb, PCV, blood pH, heterophile and H/L ratio, blood plasma glucose, total protein, A/G ratio, total lipids, cholesterol, AST, ALT, urea, and MDA of roosters exposed to HS. Instead, Bet showed partial recovery in feed intake (96.3%), ejaculate volume (94.1%), sperm concentrate/ejaculate (92.7%), total sperm/ejaculate (90.4%), semen quality factor (87.2%), seminal plasma MDA (86.5%), PA (86.5%), PI (85%), WBCs (93.2%), lymphocyte (94.8%), blood plasma albumin (87.1%), triglycerides (−10%), alkaline phosphatase (−5.8%), and creatinine (−2.2%). Bet also restored the H/L ratio to the level of the thermoneutral group, providing supporting evidence for improving welfare. These data indicate that the effect of Bet on HS-exposed animals depends on the investigated traits. In addition, Bet helps in sustaining the metabolic function when cells are under osmotic pressure, and Bet acts as a methyl donor group in protein metabolism [15]. Bet increases protein deposition in broilers [18] and ducks [9], and reduces blood urea-N by up to 47% [59], and the H/L ratio [15,48,60]. Furthermore, Bet shows an inverse relationship with obesity criteria in males [11,61].

The positive effect of Bet on reproduction and semen quality was further emphasized by the elevated feed intake, by up to 6.1% compared to the CHS group. Similarly, Singh et al. [62] found that Bet at 1.3 and 2 g/kg diet significantly increased feed intake of broiler chickens under thermal stress. In the literature, dietary Bet of 0.63 and 1.26% for boars increased sperm concentration by 6% and 13%, respectively, and total sperm output in comparison to the control group [14]. The complete recovery in fertility was validated by th increased motility, concentration, pH, and livability of the sperm in the antioxidant-fortified groups, with sperm motility being the most important indicator [1,6].

The positive effect of Bet on TAC (+32.7%) and MDA (−27.3%) in seminal plasma, while the corresponding values were +16.8 and −29.3% in blood plasma of Bet-fortified groups, suggesting that Bet is a potential antioxidant. In addition, Bet increased RBCs (17.6%), Hgb (20%), PCV (12.1%), pH (2.5%), PA (9.5%), PI (19.5%), WBCs (8%), lymphocyte (4.6%), and the H/L ratio (10.9%), demonstrating the positive impact of Bet on the health status. Similarly, Hossein and Hossein [16] reported that the H/L ratio, total IgG, and antibody titers against SRBCs were similar for the Bet-fortified group and thermoneutral group. Bet fortification decreased Urea-N levels in blood of pigs by 47%. Bet at 200 mg/kg increased survival %, immunity, and health in heat exposed chickens [15,59].

Furthermore, Bet decreased AST leakage from sperm, hepatocytes, and plasma ALP, and improved renal function (urea and creatinine). The positive effect of Bet on seminal plasma protein, globulin and total protein, blood plasma albumin, globulin and the albumin/globulin ratio indicate the boosting impact of Bet on liver function by increasing protein synthesis as a methyl group donor [10], in addition to decreasing AST and ALT [18].

Lipid metabolites (total lipids, triglycerides, cholesterol) were also improved by Bet fortification. Furthermore, MDA significantly decreased due to Bet supplementation showing the antioxidant effects of Bet. In the literature, Bet decreases meat lipid, increases the yield of breast meat [63], and improves Hgb and PCV [18]. Bet also has a sparing effect on methionine and choline [10], and thus increases choline for biosynthesis of very low-density lipoproteins, which prevent the lipid deposition via increased lipid removal from the liver [64], and regulates cholesterol in chicken [65].

It is interesting to report that Bet restored DNA in the ilium and caused a partial recovery in the jejunum DNA [66]. Bet, as a methyl donor group, increases the methionine and/or cysteine for glutathione synthesis that protects the cell from ROS and reaction metabolites, and boosts the synesthesia of DNA [17,67]. In addition, Bet is an osmolyte that stabilize proteins, cell membranes, organelles, and cells under stress [13,15]. Furthermore, recent studies by Gholami et al. [68], Nosrati et al. [69], and dos Santos et al. [70] indicate that Bet improves immunity, plasma biochemistry, and plasma osmolality of broiler chickens, suggesting its multibeneficial effects on many body functions.

In general, VE + Bet did not exceed the effect of Bet alone, even the combination of the three agents did not surpass the effect of Bet alone or Bet + VE. Body weight changes, ejaculate volume, concentration per ejaculate, total live sperm/ejaculate, and the semen quality factor of roosters supplemented with Bet + VC + VE surpassed only those of Bet + VC. Bet + VC + VE had an additive effect on seminal γ-globulin that surpassed other supplements, showing a synergetic effect over the Bet group in the current study. A similar effect was observed in H/L when the combination of the three agents exceeded the effect of Bet alone. In addition, Bet + VE or Bet + VC + VE improved plasma ALP better than Bet alone or Bet + VC, showing a synergetic effect. TAC and MDA improved due to VC and VC + VE addition compared to Bet, demonstrating an additional impact over Bet. Bet alone or Bet + VE resulted in the greatest recovery in jejunum and ileum DNA, suggesting that Bet alone is adequate for recovering the negative impact of HS on jejunum and ilium DNA. The drawback effect of Bet + VC compared to Bet alone and other combination was further validated by a drawback in the jejunum and ilium DNA recovery of VC-supplemented groups. This may adversely affect intestinal absorption capacity.

The synergetic effect of VE supplementation over Bet increased lymphocytes (cell mediated immunity), and decreased plasma albumin (nonspecific immune protein) and plasma alkaline phosphatase (index of liver function and bone mineralization), showing that a combination of Bet + VE might be beneficial for antioxidant status, and thus the animals’ immunity. The synergetic effect shown in blood biochemistry could be elucidated by the antioxidant influence of VE as confirmed herein by the increase in TAC and decrease in MDA [8]. Vitamin E is known to prevent oxidation of vitamin A and lipids. Furthermore, by increasing oxygen levels, it supports muscle growth and increases oxygen assimilation by RBCs [7]. In addition, VE acts as an essential part of the antioxidant system to prevent peroxidation of PUFA in the sperm’s membrane [71], reducing the adverse influence of corticosterone induced by stress [7,22]. VE protects cells such as macrophages, plasma cells, and lymphocytes against oxidative damage and increases the proliferation and functions of immune cells, thereby boosting animal welfare. Therefore, a 250 mg/kg VE is optimum for partly alleviating the negative influences of HS [72]. In this study, VE at 150 mg/kg feed caused a significant reduction in MDA concentrations and improved sperm motility, confirming the protective influences of VE on semen quality. This result supports VE use for treating male fertility dysfunction [25,28]. In addition, ALP, AST, and ALT were significantly decreased in males receiving 150 IU VE compared to the control roosters (15 IU VE), suggesting that 150 IU VE may be valuable for semen quality [25].

A synergetic effect of Bet + VC + VE induced a decrease in blood plasma ALP, creatinine, and MDA, and increased TAC over Bet. However, the three agents combined did not surpass the influence of Bet + VE alone. The combination of the tested agents did not exceed the effect of Bet alone on DNA repair in the intestinal segments, with Bet causing the highest and Bet + VC the lowest recovery, respectively. Jacob [73] quoted that VC enriches VE antioxidant activity by reducing tocopheroxyl radicals to their active form of VE or by sparing VE availability. In addition, Hoehler and Marquardt [30] indicated that the in vivo antioxidant influence of VE might be greater than that of VC. VE increased antibody titer and Bet + VC significantly elevated serum total protein and globulin of hens exposed to HS compared to controls [74], while decreasing serum glucose, triglyceride, HDL, and cholesterol [31]. However, Bet + VC had a similar impact on production and metabolic profiles of laying hens exposed to CHS [15].

## 5. Conclusions

Betaine at 1000 mg/kg feed for roosters had comparable effects to the combination of 1000 mg Bet with 200 mg VC and/or 150 mg E per kg diet. Therefore, Bet at 1000 mg/kg feed may be an adequate agent for compacting CHS, given the improvement of semen quality, fertility, physiological, antioxidant status, wellbeing, and intestinal DNA damage of breeder roosters, suggesting that Bet is a valuable tool for enhancing the breeding strategy of roosters in hot regions.

## Figures and Tables

**Table 1:** Ingredients and composition of the experimental diet (as fed basis).

Ingredients and Composition, g/kg	Amount
Yellow corn, ground	663.3
Soybean meal, 48% crude protein	242.0
Wheat bran	65.0
Limestone	10.0
Dicalcium phosphate	13.2
Vit + Min Premix ^1^	2.5
NaCl	2.5
DL–methionine	1.5
Calculated and determined composition	
Metabolizable energy, MJ/kg ^2^	11.98
Dry matter, g/kg ^3^	917.3
CP, g/kg^3^	179.6
Ether extract, g/kg ^3^	28.5
Crude fibre, g/kg ^3^	4.78
Methionine, g/kg ^2^	4.1
Methionine + Cysteine, TSAA, g/kg ^2^	6.70
Lysine, g/kg ^2^	8.8
Calcium, g/kg ^2^	1.10
Available P, g/kg ^2^	3.94

^1^ Vit + Min mixture provides per kilogram of diet: vitamin A, 12,000 IU; vitamin E, 10 IU; menadione, 3 mg; vitamin D3, 2200 ICU; riboflavin, 10 mg; Ca pantothenate, 10 mg; nicotinic acid, 20 mg; choline chloride, 500 mg; vitamin B12, 10 µg; vitamin B6, 1.5 mg; vitamin B1, 2.2 mg; folic acid, 1 mg; and biotin, 50 µg. Trace minerals (milligrams per kilogram of diet): Mn, 55; Zn, 50; Fe, 30; Cu, 10; Se, 0.10; antioxidants, 3 mg. ^2^ Calculated according to the National Research council (NRC) [23] tabulated values for feedstuffs.^3^ Determined values based on Reference [32].

**Table 2:** Effects of dietary betaine (Bet), with or without vitamin C (VC) and vitamin E (VE) supplementation, on semen quality in Mandarah rooster chickens reared under heat stress condition.

Parameters	Control (+)	Heat Stress Treatments					*p*-Value	SEM
		Control (−)	+ Bet	+ Bet + VC	+ Bet + VE	+ Bet + VC + VE		
Roosters performance								
Initial BW (32 wk), g	2142	2129	2115	2126	2128	2129	37.0	0.989
BW gain, g	513 ^a^	401 ^c^	490 ^a^	452 ^b^	486 ^a^	497 ^a^	11.4	0.001
Feed intake, g/bird/d	136 ^a^	123 ^c^	131 ^b^	131 ^b^	129 ^b^	130 ^b^	0.846	0.001
Semen quality characteristics								
Ejaculate volume (mL)	0.561 ^a^	0.431 ^d^	0.528 ^b,c^	0.523 ^c^	0.539 ^a,b,c^	0.549 ^a,b^	0.009	0.001
Concentrate/mL (×10^9^ sperm)	2.76 ^a^	2.11 ^b^	2.65 ^a^	2.62 ^a^	2.66 ^a^	2.70 ^a^	0.054	0.001
Concentrate/ejaculate (×10^9^ sperm)	1.55 ^a^	0.917 ^d^	1.40 ^b,c^	1.38 ^c^	1.43 ^b,c^	1.49 ^a,b^	0.039	0.001
Sperm motility (%)	90.8 ^a^	78.9 ^c^	88.7 ^a,b^	88.4 ^b^	89.1 ^a,b^	89.9 ^a,b^	0.830	0.001
Sperm livability (%)	87.2 ^a^	79.1 ^b^	86.5 ^a^	86.6 ^a^	86.9 ^a^	86.7 ^a^	0.502	0.001
Total live sperm/ejaculate (×10^9^ sperm)	1.35 ^a^	0.728 ^d^	1.22 ^b,c^	1.19 ^c^	1.25 ^b,c^	1.29 ^a,b^	0.034	0.001
Semen Ph	7.28 ^b^	7.55 ^a^	7.37 ^b^	7.29 ^b^	7.33 ^b^	7.30 ^b^	0.032	0.001
Semen quality factor	1349 ^a^	728 ^d^	1216 ^b,c^	1193 ^c^	1249 ^b,c^	1289 ^a,b^	34.3	0.001
Fertility	96.5 ^a^	79.2 ^b^	95.6 ^a^	95.6 ^a^	95.7 ^a^	96.8 ^a^	5.64	0.001
Seminal plasma constituents								
Total protein, (g/dL)	6.13 ^a^	5.48 ^b^	5.83 ^a,b^	5.80 ^a,b^	5.73 ^a,b^	5.83 ^a,b^	0.170	0.037
Albumin, (g/dL)	2.30	2.28	2.43	2.30	2.10	2.10	0.115	0.072
Globulin, (g/dL)	3.83 ^a^	3.20 ^b^	3.40 ^a,b^	3.50 ^a,b^	3.63 ^a,b^	3.73 ^a^	0.155	0.009
A/G ratio	0.604	0.714	0.717	0.659	0.582	0.567	0.050	0.022
α–globulin, (g/dL)	1.63	1.48	1.68	1.68	1.63	1.40	0.099	0.059
β–globulin, (g/dL)	1.20	1.08	1.20	1.18	1.13	1.18	0.077	0.546
γ–globulin, (g/dL)	1.00 ^a,b^	0.64 ^a,b^	0.52 ^b^	0.62 ^a,b^	0.87 ^a,b^	1.15 ^a^	0.194	0.035
AST, U/L	41.3 ^b^	51.8 ^a^	44.3 ^b^	43.8 ^b^	43.8 ^b^	42.8 ^b^	1.21	0.001
ALT, U/L	15.0 ^b^	19.8 ^a^	16.8 ^b^	16.0 ^b^	16.3 ^b^	15.8 ^b^	0.898	0.001
AST/ALT	2.77	2.65	2.66	2.76	2.68	2.75	0.170	0.967
TAC, Mmol/dL	409 ^b^	324 ^c^	430 ^a^	433 ^a^	438 ^a^	439 ^a^	4.09	0.001
MDA, Mmol/dL	0.925 ^a,b^	1.10 ^a^	0.800 ^b^	0.725 ^b^	0.700 ^b^	0.675 ^b^	0.089	0.001

BW = body weight; A/G ratio = albumin/globulin ratio; AST = aspatate amino transferase; ALT = alanine amino transferase; TAC = total antioxidant capacity; malondialdehyde (MDA); SEM = standard error of the mean. ^a–c^ means with different superscripts in the same column in similar treatment groups are significantly different.

**Table 3:** Effects of dietary betaine (Bet), with or without vitamin C (VC) and vitamin E (VE) supplementation, on hematological parameters, some immunological traits in Mandarah rooster chickens reared under heat stress condition.

Parameters	Control (+)	Heat Stress Treatments					SEM	*p*-Value
		Control (−)	+ Bet	+ Bet + VC	+ Bet + VE	+ Bet + VC + VE		
Hematological parameters and some immunological traits.								
RBCs, ×10^6^/mm^3^	1.52 ^a^	1.25 ^b^	1.47 ^a^	1.43 ^a^	1.40 ^a^	1.45 ^a^	0.067	0.001
Hgb, g/dL	10.7 ^a^	8.50 ^b^	10.2 ^a^	10.2 ^a^	9.83 ^a^	10.5 ^a^	0.585	0.012
PCV, %	32.7 ^a^	26.5 ^b^	29.7 ^a,b^	29.5 ^a,b^	30.7 ^a,b^	30.3 ^a,b^	1.54	0.015
MCV, µm^3^/red blood cell	216	214	203	206	220	212	15.4	0.892
MCH, pg/Dl	70.6	68.6	69.9	72.1	70.6	73.2	6.35	0.984
MCHC, %	32.7	32.2	34.4	34.9	32.1	34.8	2.20	0.604
Blood pH	7.54 ^b^	7.76 ^a^	7.56 ^b^	7.62 ^b^	7.62 ^b^	7.55 ^b^	0.030	0.001
PA, %	20.0 ^a^	15.8 ^c^	17.3 ^b^	17.7 ^b^	18.0 ^b^	18.0 ^b^	0.679	0.001
PI, %	1.73 ^a^	1.23 ^d^	1.47 ^bc^	1.57 ^a,b,c^	1.43 ^c^	1.65 ^a,b^	0.076	0.001
White blood cell parameters								
WBCs, ×10^3^/mm^3^	26.3 ^a^	22.5 ^c^	24.3 ^b^	24.7 ^a,b^	24.7 ^a,b^	26.0 ^a,b^	0.622	0.001
Lymphocyte, %	47.8 ^a^	43.3 ^c^	45.3 ^b^	46.2 ^a,b^	46.2 ^a,b^	47.2 ^a^	0.660	0.001
Monocyte, %	6.83	7.50	8.17	7.33	7.33	8.00	0.615	0.325
Basophil, %	0.500	0.500	0.167	0.333	0.333	0.667	0.285	0.265
Eosinophil, %	9.50	9.33	9.67	9.33	9.33	10.00	0.505	0.779
Heterophile, %	35.3 ^b^	39.3 ^a^	36.7 ^b^	36.8 ^b^	36.8 ^b^	34.2 ^b^	0.980	0.001
H/L ratio	0.739 ^b,c^	0.909 ^a^	0.810 ^b^	0.797 ^b,c^	0.797 ^b,c^	0.725 ^c^	0.028	0.001

RBC = red blood cells; PCV = packed-cell volume; Hgb = hemoglobin; MCV = mean corpuscular volume; MCH = mean corpuscular hemoglobin; MCHC= mean corpuscular hemoglobin concentration; PA = phagocytic activity; PI = phagocytic index; SEM = standard error of the mean. ^a–c^ Means with different superscripts in the same column in similar treatment groups are significantly different.

**Table 4:** Effects of dietary betaine (Bet), with or without vitamin C (VC) and vitamin E (VE) supplementation, on some blood biochemical constituents in Mandarah rooster chickens reared under heat stress condition.

Parameters	Control (+)	Heat Stress Treatments					SEM	*p*-Value
		Control (−)	+ Bet	+ Bet + VC	+ Bet + VE	+ Bet + VC + VE		
Glucose, (mg/dL)	228 ^a^	210 ^b^	223 ^a^	223 ^a^	219 ^a^	222 ^a^	2.93	0.001
Total protein, (g/dL)	5.92 ^a^	5.13 ^c^	5.62 ^a,b^	5.47 ^b,c^	5.38 ^b,c^	5.59 ^a,b^	0.140	0.001
Albumin, (g/dL)	2.64 ^a^	1.82 ^d^	2.30 ^b^	2.21 ^b,c^	2.13 ^c^	2.31 ^b^	0.054	0.001
Globulin, (g/dL)	3.28	3.32	3.32	3.26	3.26	3.28	0.173	0.998
A/G ratio	0.803 ^a^	0.550 ^c^	0.708 ^a,b^	0.680 ^a,b^	0.653 ^b,c^	0.710 ^a,b^	0.049	0.003
Total lipids, g/dL	4.42 ^b^	5.54 ^a^	4.74 ^b^	4.51 ^b^	4.56 ^b^	4.59 ^b^	0.226	0.001
Triglycerides, mg/dL	150 ^c^	175 ^a^	165 ^b^	159 ^b^	161 ^b^	158 ^b^	2.09	0.001
Cholesterol, mg/dL	136 ^b^	154 ^a^	144 ^b^	141 ^b^	138 ^b^	139 ^b^	2.71	0.001
AST, U/L	40.1 ^b^	61.5 ^a^	42.6 ^b^	42.4 ^b^	41.8 ^b^	41.5 ^b^	1.10	0.001
ALT, U/L	17.3 ^b^	21.9 ^a^	18.0 ^b^	17.7 ^b^	18.0 ^b^	17.6 ^b^	0.276	0.001
AST/ALT	2.32 ^b^	2.81 ^a^	2.36 ^b^	2.39 ^b^	2.32 ^b^	2.36 ^b^	0.065	0.001
ALP, U/l	171 ^d^	192 ^a^	181 ^b^	180 ^b^	176 ^c^	173 ^d^	1.42	0.001
Creatinine, (mg/dL)	3.19 ^c^	3.30 ^a^	3.26 ^b^	3.24 ^b,c^	3.22 ^b,c^	3.20 ^c^	0.021	0.001
Urea, (mg/dL)	3.40 ^b^	3.58 ^a^	3.43 ^b^	3.45 ^b^	3.43 ^b^	3.41 ^b^	0.040	0.001
Urea/creatinine ratio	1.07	1.08	1.05	1.06	1.07	1.07	0.012	0.450
TAC, Mmol/dL	431 ^d^	374 ^e^	437 ^c^	456 ^b^	460 ^a,b^	461 ^a^	1.92	0.001
MDA, Mmol/dL	0.915 ^b^	1.27 ^a^	0.898 ^b^	0.843 ^c^	0.818 ^c^	0.817 ^c^	0.024	0.001
DNAJ mg/g tissue	27.0 ^a^	22.8 ^d^	25.6 ^b^	24.6 ^c^	25.3 ^b^	24.8 ^c^	0.206	0.001
DNAI mg/g tissue	25.2 ^b^	20.2 ^e^	26.2 ^a^	23.3 ^d^	26.5 ^a^	24.0 ^c^	0.204	0.001

A/G ratio = albumin/globulin ratio; ALP = alkaline phosphatase; AST = aspratate amino transferase; ALT = alanine amino transferase; TAC = total antioxidant capacity; malondialdehyde (MDA). DNAJ = jejunum DNA; DNAI = ileum DNA. SEM = standard error of the mean. ^a–e^ Means with different superscripts in the same column in similar treatment groups are significantly different.

## References

[1] Khan R.U., Naz S., Nikousefat Z., Selvaggi M., Laudadio V., Tufarelli V. (2012). Effect of ascorbic acid on heat-stressed poultry. World’s Poult. Sci. J..

[2] Ayo J.O., Obidi J.A., Rekwot P.I. (2011). Effects of heat stress on the well–being, fertility, and hatchability of chickens in the northern guinea savannah zone of Nigeria: A review. ISRN Vet. Sci..

[3] Chen Z., Zhang J.R., Zhou Y.W., Liang C., Jiang Y.Y. (2015). Effect of heat stress on the pituitary and testicular development of Wenchang chicks. Arch. Anim. Breed..

[4] Pérez-Crespo M., Pintado B., Gutiérrez-Adán A. (2008). Scrotal heat stress effects on sperm viability, sperm DNA integrity, and the offspring sex ratio in mice. Mol. Reprod. Dev..

[5] Türk G., Çeribaşı A.O., Şimşek Ü.G., Çeribaşı S., Güvenç M., Kaya Ş.Ö., Çiftçi M., Sönmez M., Yüce A., Bayrakdar A. (2016). Dietary rosemary oil alleviates heat stress–induced structural and functional damage through lipid peroxidation in the testes of growing Japanese quail. Anim. Reprod. Sci..

[6] McDaniel C.D., Hood J.E., Parker H.M. (2004). An attempt at alleviating heat stress infertility in male broiler breeder chickens with dietary ascorbic acid. Int. J. Poult. Sci..

[7] Surai P.F., Kochish I.I., Fisinin V.I. (2017). Antioxidant systems in poultry biology: Nutritional modulation of vital genes. Eur. Poult. Sci..

[8] Khan R.U. (2011). Antioxidants and Poultry semen quality. World’s Poult. Sci. J..

[9] Wang Y.Z., Xu Z.R., Feng G. (2004). The effect of betaine and DL–Methionine on growth performance and cass characteristics in meat ducks. Anim. Feed Sci. Technol..

[10] Hassan R.A., Attia Y.A., El–Ganzory E.H. (2005). Growth, carcass quality, and blood serum constituents of slow growth chicks as affected by betaine Additions to diets containing 1. Different levels of choline. Int. J. Poult. Sci..

[11] Gao X., Randell E., Zhou H., Sun G. (2018). Higher serum choline and betaine levels are associated with better body composition in male but not female population. PLoS ONE.

[12] Attia Y.A., Hassan R.A., Shehatta M.H., Abd El-Hady S.B. (2005). Growth, carcass quality and blood serum constituents of slow growth chicks as affected by betaine additions to diets containing 2. Different levels of methionine. Int. J. Poult. Sci..

[13] Ahmed Mervat M.N., Zienhom S.H., Ismail Ahmed A., Abdel-Wareth A. (2018). Application of betaine as feed additives in poultry nutrition-a review. J. Exp. Appl. Anim. Sci..

[14] Cabezón F.A., Stewart K.R., Schinckel A.P., Barnes W., Boyd R.D., Wilcock P., Woodliff J. (2016). Effect of natural betaine on estimates of semen quality in mature AI boars during summer heat stress. Anim. Reprod. Sci..

[15] Attia Y.A., Abd-El-Hamid A.E., Abedalla A.A., Berika M.A., Al-Harthi M.A., Kucuk O., Sahin K., Abou–Shehema B.M. (2016). Laying performance, digestibility and plasma hormones in laying hens exposed to chronic heat stress as affected by betaine, vitamin C, and/or vitamin E supplementation. Springerplus.

[16] Hossein A.S., Hossein A.G. (2016). Alleviation of chronic heat stress in broilers by dietary supplementation of betaine and turmeric rhizome powder: Dynamics of performance, leukocyte profile, humoral immunity, and antioxidant status. Trop. Anim. Health Prod..

[17] Alirezaei M., Jelodar G., Ghayemi Z. (2012). Antioxidant defense of betaine against oxidative stress induced by ethanol in the rat testes. Int. J. Pept. Res. Ther..

[18] Attia Y.A., Hassan R.A., Qota E.M. (2009). Recovery from adverse effects of heat stress on slow-growing chicks in the tropics 1: Effect of ascorbic acid and different levels of betaine. Trop. Anim. Health Prod..

[19] McDowell L.R. (1989). Vitamins in Animal Nutrition.

[20] Klasing K.C. (1998). Nutritional modulation of resistance to infectious diseases. Poult. Sci..

[21] Mckee J.S., Harrison P.C. (1995). Effects of supplemental ascorbic acid on the performance of broiler breeders following exposure to an elevated environmental temperature. Poult. Sci..

[22] Metwally M.A. (2005). Efficacy of different dietary levels of antioxidants vitamin C, E and their combination on the performance of heat-stressed laying hens. Egypt. Poult. Sci. J..

[23] National Research Council (NRC) (1994). Nutrient Requirement of Poultry.

[24] Surai P.F. (1999). Vitamin E in avian reproduction. Poult. Avi. Biol. Rev..

[25] Biswas A., Mohan J., Sastry K.V.H., Tyagi J.S. (2007). Effect of dietary vitamin E on the cloacal gland, foam and semen characteristics of male Japanese quail. Theriogenology.

[26] Ebied T.A. (2012). Vitamin E and organic selenium enhances the antioxidative status and quality of chicken semen under high ambient temperature. Br. Poult. Sci..

[27] Echeverria S., Santos R., Centurion F., Ake R., Alfaro M., Rodriguez J. (2009). Effects of dietary selenium and vitamin E on semen quality and sperm morphology of young boars during warm and fresh season. J. Anim. Vet. Adv..

[28] Keskes-Ammar L., Feki-Chakroun N., Rebai T., Sahnoun Z., Ghozzi H., Hammami S., Zghal K., Fki H., Damak J., Bahloul A. (2003). Sperm oxidative stress and the effect of an oral vitamin E and selenium supplement on semen quality in infertile men. J. Reprod. Syst..

[29] Traber M.G., Atkinson J. (2007). Vitamin E, antioxidant and nothing more. Free Radic. Biol. Med..

[30] Hoehler D., Marquardt R.R. (1996). Influence of Vitamin E and C on the toxic effects of ochratoxin A and T–2 Toxin in chicks. Poult. Sci..

[31] Ezzat W., Shoeib M.S., Mousa S.M.M., Bealish A.M.A., Ibrahiem Z.A. (2011). Impact of betaine, vitamin C and folic acid supplementation to the diet on productive and reproductive performance of Matrouh poultry strain under Egyptian summer condition. Egypt. Poult. Sci. J..

[32] Association of official analytical chemists (2004). Official Methods of Analysis.

[33] Attia Y.A., Abd El-Hamid E.A., El-Hanoun A.M., Al-Harthi M.A., Mansour G.M., Abdella M.M. (2015). Responses of the fertility, semen quality, blood constituents, immunity and antioxidant status of rabbit bucks to type and magnetizing of water. Ann. Anim. Sci..

[34] Bootwalla S.M., Miles R.D. (1992). Development of diluents for domestic fowl semen. World’s Poult. Sci. J..

[35] Yan F., Kersey J.H., Fritts C.A., Waldroup P.W., Stilborn H.L., Crumm R.C., Rice D.W. (1972). DGKC: Recommendations of the German Society for Clinical Chemistry, Z. Klin. Chim. Clin. Biochem..

[36] Bianchi A.T.J., Moonen–Leusen H.W.M., van der Heijden P.J., Bokhout B.A. (1995). The use of a double antibody sandwich ELISA and monoclonal antibodies for the assessment of porcine IgM, IgG, and IgA concentrations. Vet. Immunol. Immunopathol..

[37] Tietz N.W. (1982). Fundamental of Clinical Chemistry 3^rd^ Edition.

[38] Helper O.E. (1966). Manual of Clinical Laboratory Methods.

[39] Hawkeye C.M., Dennett T.B. (1989). A Color Atlas of Comparative Veterinary Hematology.

[40] Wintrobe M.M. (1965). Clinical Hematology.

[41] Kawahara E., Ueda T., Nomura S. (1991). In vitro phagocytic activity of white spotted shark cells after injection with Aermonassalmonicida extracellular products. GyobokenkyuJpn.

[42] Lucky Z. (1977). Methods for the Diagnosis of Fish Diseases.

[43] Sambrook J., Fristsch E.F., Maniatis M. (1989). Molecular Cloning A Laboratory Manual.

[44] Abdel-Fattah A.F., Mohamed E.H., Ramadan G. (1995). Effect of thymus extract on immunologic reactively of chicken vaccinated with infectious bursal disease virus. J. Vet. Med. Sci..

[45] Charles P., Ledouix L. (1970). Isolation of Deoxyribonucleic Acid: Uptake of Informative Moleculas by Living Cells.

[46] (2007). Statistical Analyses Software, SAS, Institute: SAS User’s Guide.

[47] Lentfer T.L., Pendl H., Gebhardt-Henrich S.G., Fröhlich E.K., Von Borell E. (2015). H/L ratio as a measurement of stress in laying hens-methodology and reliability. Br. Poult Sci..

[48] Attia Y.A., Hassan R.A., Tag El-Din A.E., Abou-Shehema B.M. (2011). Effect of ascorbic acid or increasing metabolizable energy level with or without supplementation of some essential amino acids on productive and physiological traits of slow-growing chicks exposed to chronic heat stress. J. Anim. Phys. Anim. Nutr..

[49] Sahin K., Sahin N., Yaralioglu S. (2002). Effects of vitamin C and vitamin E on lipid peroxidation, blood serum metabolites, and mineral concentrations of laying hens reared at high ambient temperature. Biol. Trace Elem. Res..

[50] Zeng T., Li J.J., Wang D.Q., Li G.Q., Wang G.L., Lu L.Z. (2014). Effects of heat stress on antioxidant defense system, inflammatory injury, and heat shock proteins of Muscovy and Pekin ducks: Evidence for differential thermal sensitivities. Cell Stress Chaperones.

[51] Toyomizu M., Tokuda M., Mujahid A., Akiba Y. (2005). Progressive alteration to core temperature, respiration and blood acid-base balance in broiler chickens exposed to acute heat stress. J. Poult. Sci..

[52] Daghir N.J. (2008). Poultry Production in Hot Climates.

[53] Habashy W.S., Milfort M.C., Adomako K., Attia Y.A., Rekaya R., Aggrey S.E. (2017). Effect of heat stress on amino acid digestibility and transporters in meat–type chickens. Poult. Sci..

[54] Habashy W.S., Milfort M.C., Fuller A.L., Attia Y.A., Rekaya R., Aggrey S.E. (2017). Effect of heat stress on protein utilization and nutrient transporters in meat–type chickens. Int. J. Biometeorol..

[55] Stone B.A., Seamark R.F. (1984). Effects of acute and chronic testicular hyperthermia on levels of testosterone and corticosteroids in plasma of boars. Anim. Reprod. Sci..

[56] Peña S., Gummow B., Parker A.J., Paris D.B.B.P. (2016). Revisiting summer infertility in the pig: Could heat stress–induced sperm DNA damage negatively affect early embryo development?. Anim. Reprod. Sci..

[57] Zheng W.W., Song G., Wang Q.L., Liu S.W., Zhu X.L., Deng S.M., Zhong A., Tan Y.M., Tan Y. (2018). Sperm DNA damage has a negative effect on early embryonic development following in vitro fertilization. Asian J. Androl..

[58] Sutovsky P., Aarabi M., Miranda-Vizuete A., Oko R. (2015). Negative biomarker based male fertility evaluation: Sperm phenotypes associated with molecular-level anomalies. Asian J. Androl..

[59] Eklund M., Bauer E., Wamatu J., Mosenthinm R. (2005). Potential nutritional and physiological functions of betaine in livestock. Nutr. Res. Rev..

[60] Attia Y.A., Böhmer Barbara M., Roth-Maier D.A. (2006). Responses of broiler chicks raised under constant relatively high ambient temperature to enzymes, amino acid supplementations, or diet density. Arch. Geflügelk..

[61] Attia Y.A., Burke W.H., Yamani Y.A., Jensen L.S. (1995). Energy allotments and performance of broiler breeders 1. Males. Poult. Sci..

[62] Singh A.K., Ghosh T.K., Creswell D.C., Haldar S. (2015). Effects of supplementation of betaine hydrochloride on physiological performances of broilers exposed to thermal stress. Open Access Anim. Phys..

[63] Metzler-Zebeli B.U., Eklund M., Mosenthin R. (2009). Impact of osmoregulatory and methyl donor functions of betaine on intestinal health and performance in poultry. World’s Poult. Sci. J..

[64] Yao Z., Vance D. (1989). Head group specificity in the requirement of phosphatidylcholine biosynthesis for very low density lipoprotein secretion from cultured hepatocytes. J. Biol. Chem..

[65] Yun H., Sun Q., Li X., Wang M., Cai D., Li X., Zhao R. (2015). In Ovo injection of betaine affects hepatic cholesterol metabolism through epigenetic gene regulation in newly hatched chicks. PLoS ONE.

[66] Zabrodina V.V., Shreder O.V., Shreder E.D., Durnev A.D. (2016). Effect of afobazole and betaine on cognitive disorders in the offspring of rats with streptozotocin–induced diabetes and their relationship with DNA damage. Bull. Exp. Biol. Med..

[67] Zhang M., Hong Z., Li H., Lai F., Li X.F., Tang Y., Tian M., Hui W. (2016). Antioxidant mechanism of betaine without free radicals scavenging ability. J Agric. Food Chem..

[68] Gholami J., Qotbi A.A.A., Seidavi A.R., Meluzzi A., Tavaniello S., Maiorano G. (2015). Effects of in ovo administration of betaine and choline on atchability results, growth and carcass characteristics and immune response of broiler chickens. Ital. J. Anim. Sci..

[69] Nosrati M., Javandel F., Camacho L.M., Khusro A., Cipriano M., Seidavi A.R., Salem A.Z.M. (2017). The effects of antibiotic, probiotic, organic acid, vitamin C, and Echinacea purpurea extract on performance, carcass characteristics, blood chemistry, microbiota, and immunity of broiler chickens. J. Appl. Poult. Res..

[70] Dos Santos T.T., Baal S.C.S., Lee S.A., e Silva F.R.O., Scheraiber M., da Silva A.V.F. (2019). Influence of dietary fibre and betaine on mucus production and digesta and plasma osmolality of broiler chicks from hatch to 14 days of age. Livest. Sci..

[71] Tran L.V., Malla B.A., Kumar S.A.K. (2017). Polyunsaturated fatty acids in male ruminant reproduction—A Review. Asian Aust. J. Anim. Sci..

[72] Bollinger-Lee S., Williams P.E., Whitehead C.C. (1999). Optimal dietary concentration of vitamin E for alleviating the effect of heat stress on egg production in laying hens. Br. Poult. Sci..

[73] Jacob R.A. (1995). The integrated antioxidant system. Nutr. Res..

[74] Mohiti-Asli M., Shariatmadari F., Lotfollahian H. (2010). The influence of dietary vitamin E and selenium on egg production parameters, serum and yolk cholesterol and antibody response of laying hen exposed to high environmental temperature. Arch. Geflügelk..

